# Disparities of demographics, clinical characteristics, and hospital outcomes of AMI pilgrims vs non-pilgrims—tertiary center experience

**DOI:** 10.1186/s43044-020-00068-y

**Published:** 2020-05-29

**Authors:** Sheeren Khaled, Walaa Eldeen Ahmed, Ghada Shalaby, Hadeel Alqasimi, Rahaf Abu Ruzaizah, Mryam Haddad, Mroj Alsabri, Seham Almalki, Heba Kufiah, Fatma Aboul Elnein, Najeeb Jaha

**Affiliations:** 1 Banha University, Banha, Egypt; 2 King Abdullah Medical City, Muzdallfa Road, Makkah, Saudi Arabia; 3 Zagazige University, Makkah, Egypt; 4 Collage Of Medicine, Umm Al Qura University, Makkah City, Makkah 24353 Saudi Arabia; 5 Collage of Medicine, Taif University, Taif City, Makkah 26561 Saudi Arabia; 6 Alexandria university, Alexandria, Egypt

## Abstract

**Background:**

Acute myocardial infarction (AMI) is usually caused by rupture of an atherosclerotic plaque leading to thrombotic occlusion of a coronary artery. Cardiovascular disease has recently emerged as the leading cause of death during hajj. Our aim is to demonstrate the AMI pilgrim’s related disparities and comparing them to non-pilgrim patients.

**Result:**

Out of 3044 of patients presented with AMI from January 2016 to August 2019, 1008 (33%) were pilgrims. They were older in age (*P* < 0.001) and showed significantly lower rates cardiovascular risk factors (*P* < 0.001 for DM, smoking, and obesity). Pilgrims were also less likely to receive thrombolytic therapy (*P* < 0.001), show lower rate of late AMI presentation (*P* < 0.001), develop more LV dysfunction post AMI (*P* < 0.001), and have critical CAD anatomy in their coronary angiography (*P* < 0.001 for MVD and = 0.02 for LM disease) compared to non-pilgrim AMI patients. Despite AMI pilgrims recorded higher rate of primary percutaneous coronary intervention (PPCI) procedures, they still showed poor hospital outcomes (*P* < 0.001, 0.004, < 0.001, 0.05, and 0.001, respectively for pulmonary edema, cardiogenic shock, mechanical ventilation, cardiac arrest, and in-hospital mortality, respectively). Being a pilgrim and presence of significant left ventricular systolic dysfunction, post AMI was the two independent predictors of mortality among our studied patients (*P* = 0.005 and 0.001, respectively).

**Conclusion:**

Although AMI pilgrims had less cardiovascular risk factors and they were early revascularized, they showed higher rates of post myocardial infarction complication and poor hospital outcomes. Implementation of pre-hajj screening, awareness and education programs, and primary and secondary preventive measures should be taken in to consideration to improve AMI pilgrim’s outcome.

## Background

Acute myocardial infarction (AMI) is usually caused by interaction of lipoprotein retention, inflammatory process, and rupture of an atherosclerotic plaque leading to thrombotic occlusion of a coronary artery. It is classified into ST-elevation myocardial infarction (STEMI) and non ST-elevation myocardial infarction (NSTEMI). These patients are generally treated with combination of medical therapy and revascularization with percutaneous coronary intervention (PCI) or coronary artery bypass grafting (CABG) [1, 2].

Hajj is a great event, and it is one of the five Islamic pillars. Millions of pilgrims from different countries of the world come to the kingdom of Saudi Arabia for performing hajj. The overcrowding, hot climate, and huge physical stress expose the pilgrims to many health hazards. Cardiovascular disease has recently emerged as the leading cause of death during hajj [3, 4].

Our center is the only cardiac center in the region providing tertiary care facility including PCI and CABG and hence receives all AMI patients deemed suitable for invasive assessment and management serving huge number of hajj patients with distinct racial, socioeconomic, and demographic characteristics.

There are no studies done before comparing the AMI clinical and in-hospital outcome between pilgrims and non-pilgrims. Our aim is to demonstrate the possible disparities of demographics, clinical data, and in-hospital outcomes of AMI patients admitted to our specialized cardiac center in Makkah region, Saudi Arabia.

## Methods

It is a retrospectively collected data of the prospective single-center study that included 3044 patients who were admitted and underwent coronary angiography at the cardiac center—King Abdullah Medical City (KAMC)—from January 2016 till August 2019.

### Inclusion criteria

All patients admitted to our cardiac center with established diagnosis of AMI (based on clinical features, including electrocardiographic (ECG) findings, elevated values of biochemical markers (biomarkers) of myocardial necrosis, and by imaging [5]) and underwent coronary angiography and revascularization (including primary PCI, elective PCI, and rescue PCI) from 2016 to 2019 including hajj seasons.

### Exclusion criteria

Patients with AMI treated conservatively and coronary angiography was not done because of preference of the patient or due to other reasons.

Our study is designed to be a part of the standards of patient’s care, to investigate and improve quality of AMI management and outcomes among diverse population, general and cath consents were taken, and has received approval of the ethics committee/institutional review board of our institution (KAMC).

All demographic data (age, gender, nationality, BMI, and status (residence/hajj)), risk factors (DM [6], HTN [6], smoking, dyslipidemia [6], presence of chronic kidney disease, old cerebrovascular accidents (CVA) ischemic heart disease (IHD), and previous PCI/CABG), clinical presentation (location of AMI), laboratory results (hemoglobin (HB), BUN, creatinine, HB A1c, and troponin (1st and peak quantitative values)), coronary angiography findings (access site (femoral/radial), LM (left main) disease, 3 vessel CAD (coronary artery disease), PCI artery, thrombus aspiration and tirofiban/Aggrastat use), outcomes (in hospital death, length of stay (LOS), LVEF [7] (left ventricular ejection fraction), pulmonary edema, cardiac arrest, cardiogenic shock, history of mechanical ventilation (MV), and history of LVT (left ventricular thrombus)) for each patient were recorded. Data were collected from cath lab records and their case notes including electronic records, echocardiographic, and coronary angiographic reports.

Late presentation was defined as a time interval until hospital arrival of more than 6 h after the onset of the symptom [8].

Door to balloon time was defined as 90 min or less for patients undergoing primary percutaneous coronary intervention (PCI) according to guidelines [9].

### Statistical analysis

Statistical analysis was performed by the use of the SPSS software package (SPSS Inc.; Chicago, IL), version 21.0. Data are presented as mean ± SD for continues data and percentage for categorical variables. Univariate analysis were done using *t*-test or chi-squared test for continues and categorical data, respectively. Cox-regression analysis was also performed. For all analyses, a *P* value < 0.05 was considered significant and not significant if it is > 0.05.

## Results

**Fig. 1:**
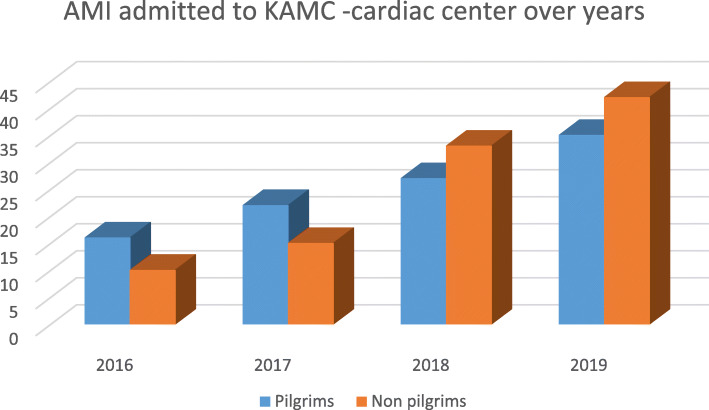
AMI pilgrims and non-pilgrims admission to KAMC from 2016 to 2019

A total of 3044 AMI patients were admitted to our cardiac center from January 2016 till August 2019 (Fig. 1) and were classified in to two groups: group I, 1008 (33%) pilgrim patients; group II, 2036 (67%) non-pilgrim patients. We categorized our data into three main categories: baseline characteristics, clinical measures, and in-hospital outcome data.

### Baseline characteristics

AMI pilgrims were older in age (58.10 ± 10.88 vs 55.13 ± 12.07; *P* < 0.001), showed less male gender (78% vs 87%; *P* < 0.001) and more south Asian population compared to group II patients (49% vs 20%; *P* < 0.001). Patients of group II had significantly higher prevalence history of DM, smoking, and obesity compared to group I (57%, 40%, and 32% vs 48%, 20%, and 23%, respectively; *P* < 0.001). There were no observed significant differences between the two groups regarding the rates of HTN, hyperlipidemia, IHD, and previous coronary revascularization. Regarding to laboratory findings, AMI pilgrim patients had significantly lower HB level on presentation with higher BUN value suggestive of their dehydration compared to group II patients (*P* < 0.001) (Table 1).

**Table 1:** Baseline demographic data of pilgrim and non-pilgrim AMI patients

Variable	Pilgrims, ***N*** = 1008 (33%)	Non-pilgrims, ***N*** = 2036 (67%)	***P*** value
**Age (years)**	58.10 ± 10.88	55.13 ± 12.07	< 0.001
**Male gender**	789 (78%)	1763 (87%)	< 0.001
**South Asian**	487 (49%)	399 (20%)	< 0.001
**Middle Eastern**	521 (51%)	1637 (80%)	< 0.001
**BMI (kg/m**^**2**^**)**	27.07 ± 4.83	28.18 ± 5.31	< 0.001
**HBA1-C (mmol/mol)**	7.40 ± 2.74	7.79 ± 2.95	0.002
**Admission HB level (g/dL)**	13.32 ± 1.96	14.08 ± 2.02	< 0.001
**Platelets count (× 10**^**9**^**/L)**	219 ± 18	210 ± 14	NS
**Troponin 1st (ng/mL)**	91.78 ± 201	91.32 ± 273	NS
**Troponin peak (ng/mL)**	100.19 ± 157	94.07 ± 258	NS
**BUN (mmol/L)**	18.29 ± 10.57	16.85 ± 9.31	< 0.001
**Serum creatinine (mg/dL)**	1.35 ± 4.79	1.21 ± 2.12	NS
**DM**	488 (48%)	1168 (57%)	< 0.001
**Smoking**	204 (20%)	806 (40%)	< 0.001
**Obesity (BMI > 30 kg/m**^**2**^**)**	220 (23%)	637 (32%)	< 0.001
**HTN**	536 (53%)	1096 (54%)	NS
**DLP**	126 (13%)	297 (15%)	NS
**CVA**	15 (1.5%)	62 (3%)	0.01
**IHD**	187 (16%)	428 (21%)	NS
**OLD PCI/CABG**	67 (7%)	145 (7%)	NS
**STEMI, anterior**	551 (55%)	1101 (54%)	
**Inferior**	411 (41%)	841 (41%)	NS
**Other**	46 (4%)	94 (5%)	
**Thrombolytic therapy**	99 (10%)	468 (23%)	< 0.001
**Late presentation**	90 (9%)	285 (14%)	< 0.001
**Tirofiban**	257 (26%)	484 (24%)	NS
**Thrombus aspiration**	134 (13%)	242 (12%)	NS

*BMI* body mass index, *CABG* coronary artery bypass grafting, *CVA* cerebrovascular accidents, *DLP* dyslipidemia, *DM* diabetes mellitus, *HB* hemoglobin, *HBA1C* glycosylated hemoglobin, *IHD* ischemic heart disease, *PCI* percutaneous coronary intervention

### Clinical measures

With regard to the type of STEMI, intensive anti-ischemic treatment (use of thrombus aspiration during coronary angiogram and tirofiban), there were no significant differences between the two groups. In Table 1, pilgrims were less likely to receive thrombolytic therapy and showed lower rate of late AMI presentation compared to non-pilgrims (*P* < 0.001). Moreover, post AMI-LVEF was recorded lower among pilgrim patients compared to non-pilgrims (39.71% ± 11.45% vs 41.46% ± 10.58%; *P* < 0.001). Also, pilgrim patients had higher prevalence of three vessel disease (3VD) and left main (LM) disease compared to non-pilgrims (19% and 4% vs 14% and 2%; *P* < 0.001 and 0.02, respectively). Primary percutaneous coronary intervention (PPCI) was utilized relatively higher by pilgrims, and the recorded door to balloon time < 90 min was achieved higher by AMI pilgrim patients compared to non-pilgrims (Table 2).

**Table 2:** Echocardiography and coronary angiography of pilgrim and non-pilgrim AMI patients

Variable	Pilgrims, ***N*** = 1008 (33%)	Non-pilgrims, ***N*** = 2036 (67%)	***P*** value
**Post AMI LVEF%**	39.71% ± 11.45%	41.46% ± 10.58%	< 0.001
**Trans-radial axis**	725 (72%)	1626 (80%)	0.018
**DBT < 90 min for PPCI**	295 (29%)	507(25%)	NS
**LM disease**	39 (4%)	49 (2%)	0.02
**3VD**	191 (19%)	284 (14%)	< 0.001
**PPCI**	604 (60%)	1180 (58%)	NS

*AMI* acute myocardial infarction, *DBT* door to balloon time, *LM* left main, *LVEF* left ventricular ejection fraction, *PCI* percutaneous coronary intervention, *PPCI* primary percutaneous coronary intervention, *3VD* three vessel disease

### In-hospital outcomes

AMI pilgrims showed higher rates of post myocardial infarction complications (*P* < 0.001, 0.004, 0.001, < 0.001, and 0.05, respectively, for pulmonary edema, cardiogenic shock, severe LV dysfunction, mechanical ventilation, and cardiac arrest, respectively). The total in-hospital mortality was 3%, and it is recorded higher among pilgrim patients compared to the second group (4.5% vs 2%; *P* = 0.001). Incidence of both left ventricular thrombus formation post AMI and major bleeding did not differ between the two groups (Table 3).

**Table 3:** Hospital outcome data of pilgrim and non-pilgrim AMI patients

Variable	Pilgrims, ***N*** = 1008 (33%)	Non-pilgrims, ***N*** = 2036 (67%)	***P*** value
**Sever LVD (LVEF < 30%)**	243 (24%)	391 (19%)	< 0.001
**LOS (days)**	4.87 ± 6.55	6.07 ± 8.48	< 0.001
**MV**	81 (8%)	64 (3%)	< 0.001
**Pulmonary edema**	59 (6%)	48 (2%)	< 0.001
**Cardiogenic shock**	58 (6%)	70 (3%)	0.004
**Cardiac arrest**	66 (7%)	98 (5%)	0.05
**In-hospital mortality**	45 (4.5%)	46 (2%)	0.001
**Major bleeding (HB drop > 3 g)**	50 (5%)	122 (6%)	NS
**LVT**	80 (8%)	183(9%)	NS

*HB* hemoglobin, *LOS* length of stay, *LVD* left ventricular dysfunction, *LVT* left ventricular thrombus, *MV* mechanical ventilation

**Fig. 2:**
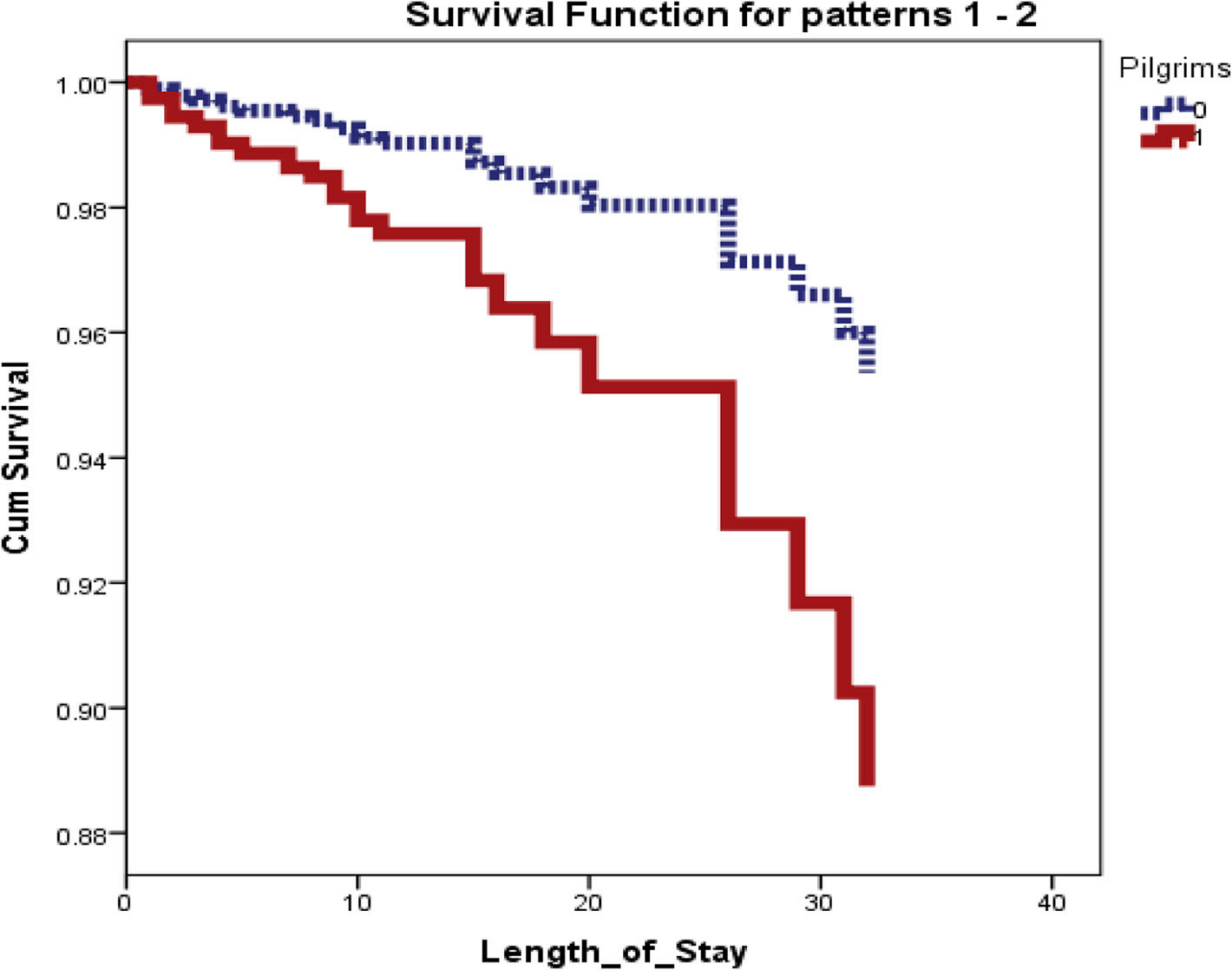
Regression analysis—pilgrims are independent predictor of the mortality

Being a pilgrim and presence of significant left ventricular dysfunction post-acute myocardial infarction were found the two independent predictors of in-hospital death post AMI in the studied patients; however age, DM, HTN, LM disease, and MVD were all not found to be independent predictors of death (Table 4 and Fig. 2).

**Table 4:** Cox regression analysis of in-hospital mortality

Variable	B	SE	Wald	df	Sig.	Exp(B)	95.0% CI for Exp(B)	
							Lower	Upper
**Age**	.028	.015	3.561	1	.059	1.028	.999	1.058
**Male**	− .713	.387	3.398	1	.065	.490	.230	1.046
**DM**	.575	.379	2.301	1	.129	1.778	.845	3.738
**HTN**	− .428	.349	1.506	1	.220	.652	.329	1.292
**LM disease**	.548	.517	1.123	1	.289	1.730	.628	4.765
**MVD**	.338	.361	.873	1	.350	1.402	.690	2.847
**LVD**	− .071	.014	26.635	1	.000	.932	.907	.957
**Pilgrims**	.922	.328	7.892	1	.005	2.514	1.321	4.784

*DM* diabetes mellitus, *HTN* hypertension, *LM* left main, *MVD* multi vessel disease, *LVD* left ventricular dysfunction

**Fig. 3:**
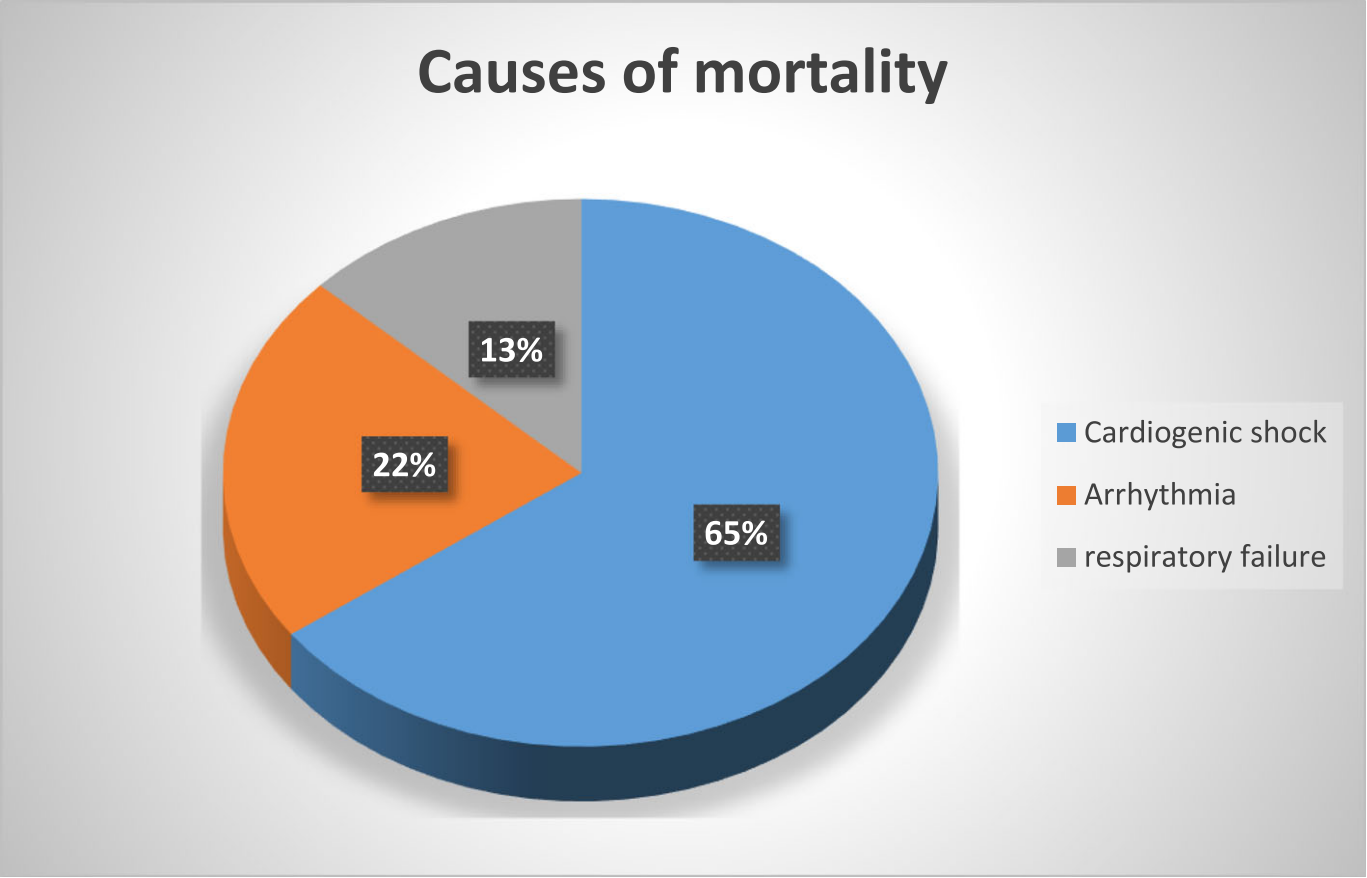
Causes of mortality

**Fig. 4:**
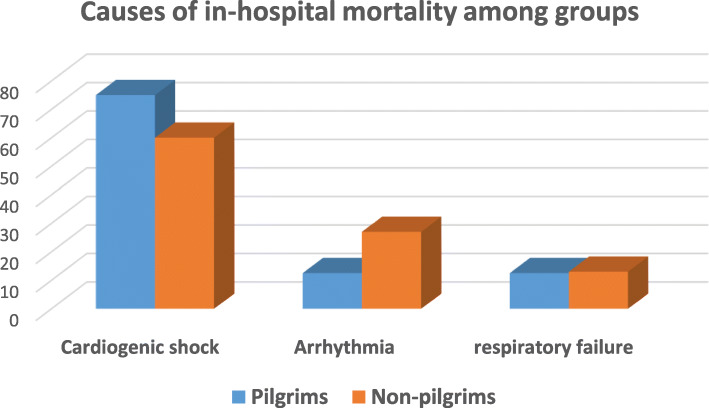
Causes of in-hospital death in pilgrims and non-pilgrims

Cardiogenic shock (65%), arrhythmias (22%), and respiratory failure (13%) were the most common causes of death among our studied AMI patients (75%, 12.5%, and 12.5% vs 60%, 27%, and 13%, respectively, for pilgrims and non-pilgrims) (Figs. 3 and 4).

## Discussion

The location of our tertiary cardiac center in the holy city of Makkah near to Haram and holy sites of Mena, Arafat, and Mouzdalifa is unique as it received all AMI indicated for interventions including huge number of hajj patients increasing yearly; hence, this created and raised the idea for the current study. No prior study conducted in Saudi Arabia comparing pilgrim vs non-pilgrim AMI patients regarding clinical characteristics, management, and outcomes.

Three thousand and forty-four patients with diagnosis of acute myocardial infarction were referred and admitted to our cardiac center from 2016 till 2019 for further revascularization. During the four hajj seasons (2016–2019), one thousand and eight pilgrim AMI patients were admitted and treated in our center. Pilgrim patients presented with acute myocardial infarction were elderly compared to non-pilgrims, and this is expected, as these are people in such age category who come for the hajj pilgrimage after finishing all their essential responsibilities. This is prescribed in previous few studies, which conducted to assess pattern of CVD among pilgrims [10, 11]. Interestingly, those hajj patients in spite of being elderly, they had lower cardiovascular risk profile compared to residence in Saudi Arabia. Different genetic, racial, environmental, and cultural backgrounds of those populations who came from variable places might explain this. Also, AMI pilgrim patients had significant values of laboratory markers suggestive of their dehydration and hyper-osmolarity compared to non-pilgrims, and this is addressed by our group in a different study [12].

Moreover, from our interesting findings in the current study, those AMI pilgrims were less likely to receive thrombolytic therapy and showed lower rate of late presentation compared to non-pilgrims. This could be explained by during hajj, cath lab work full capacity 24 h, 12 h shift day same as night; however, residence of Makkah sometimes come from remote area that managed by drip and shift strategy while pilgrims are mostly within Makkah city. Also, those AMI hajj patients had higher prevalence of severe coronary artery disease (multi-vessel CAD and LM disease) compared to residence, and in spite of being early revascularized with PPCI, they still showed significant LV dysfunction in their echocardiogram post MI. This could be explained by many factors including genetic variation, degree of atherosclerosis, different thrombotic activity, distribution of collateral circulation, and severe physical and emotional stress they had been developed during hajj. Moreover, the utilization rate of PPCI was recorded relatively higher among pilgrim patients with higher achieved door to balloon time < 90 min. There are several factors could explain the significant improvement of the service provided to AMI hajj patients. First, the great effort and support of the government of Saudi Arabia through the ministry of health and hajj committee to implement the program of increasing hajj manpower included interventionist cardiologist working at our center during hajj seasons. Second, raise number of the working cath labs. Third, well establishment of organized network for urgent transport of the AMI hajj patients from all Al-Mashaer, primary, and secondary hospitals to our tertiary hospital; this includes air medevac transportation. Fourth, proper preparation of all Makkah hospitals with emergency situations and to provide short-term hospitalization services with immediate transfer to our center for intervention and urgent revascularization.

Surprisingly, those AMI hajj patients who were early revascularized with PPCI, they showed significant higher rates of post AMI complications and poor outcomes. With respect of the mortality, the over all in-hospital mortality rate among our studied AMI patients was recorded (3%), and it was comparable to which reported by a recent AMI registry in Saudi Arabia [13]; however, it is still higher among pilgrim patients compared to non-pilgrims. Moreover, it was found that being a pilgrim would double the risk of death once got AMI. These poor in-hospital outcomes among pilgrim patients could be explained by several factors: being elderly, had critical CAD anatomy (multi-vessel CAD and LM disease), were exposed to great dehydration, severe physical and emotional stress that all might lead to huge deterioration, and adverse impact LV function and hence poor outcomes. Also, in spite of having more complication, pilgrims showed shorter length of hospital stay compared to non-pilgrims, and this might be related to many factors. First, many of them signed discharge against medical advice and went to complete their hajj and then back to their own countries. Second, referring back to their primary hospitals after revascularization to complete their hospital course (due to high need of beds during hajj season to compensate more patients who need coronary angiography). Third, some of them are taken by their groups to complete their medical care under their own responsibilities.

Unlike AMI pilgrims, non-pilgrim patients who presented with myocardial infarctions were younger in age with higher prevalence of cardiovascular risk factors. Our findings consisted with a recent multi-center registry study in 50 hospitals from various health care sectors in Saudi Arabia were conducted for AMI to evaluate the clinical characteristics, management, and outcomes of a representative sample of patients with acute myocardial infarction (AMI) in Saudi Arabia. It concluded that AMI patients in Saudi Arabia presented at relatively young age and had a high prevalence of CAD risk factors [13]. This is also similar to results from prior ACS registries in Saudi Arabia [14] and mentioned by several other studies [15–17].

Interestingly that all our AMI population had proper access to primary PCI with higher recorded rates than reported in the previous studies [13, 18–20]. This is explained by the unique and valuable location of our center in the region with 24/7 acute interventional facilities, receive most of MI patients with significantly improvement in the communication, and transportation of the patients from all peripheral hospitals. Moreover, the recorded rate of primary PCIs utilization is relatively higher among pilgrim patients. This is because of the above mentioned increased proper facility services provided during the hajj seasons, and actually this is considered as unique issue provided by our center for early revascularization to those hujaj who were likely in need to complete the hajj and return back to their countries. The in-hospital outcome measures including the mortality rate were better in non-hajj compared to AMI pilgrims, and this could be explained by being younger at age, had better LVEF post AMI, and less critical CAD anatomy compared to pilgrim patients.

Finally, cardiogenic shock, arrhythmias, and respiratory failure were the most common causes of death among our patients with observed less recorded arrhythmic death among pilgrims compared to non-pilgrims. This is unclear but might be explained by different genetic factors, being early revascularized with PPCI (less myocardial necrosis and scare formation which predispose to arrhythmias) and early discharged from the hospital (late arrhythmias might be not detected).

In conclusion, hajj is a major annual mass gathering of millions of people from all over the world, with different cultures, socioeconomic levels, knowledge, attitudes, and most importantly health statuses [21]. The causes of morbidities and mortalities are varied [22]. Current study provides detailed information about cardiovascular risk profile, clinical characteristics, hospital course, and short-term outcomes of pilgrim patients admitted with AMI.

## Recommendation

Identification and awareness of the causes and clinical characters of those patients might reduce the hazards. This also highlights future strategic planning, motivations of more implementation of pre-hajj screening and education programs, and encourage the multidisplinary and multicentric cooperation in proper serving those high-risk pilgrims. Moreover, this study also refers to the need to develop optimal secondary prevention measures to be applied after hospital discharge as it was found that AMI residence non-pilgrim patients were younger at age and had multiple cardiovascular risk factors.

## Limitation

The number of enrolled patients is limited due to the nature of single center study and selection of AMI patients only. Moreover, no follow-up data or long-term outcomes and that is because one group of our study was pilgrims; they were going back to their countries after doing hajj, and hence no follow-up were available. We hope to reduce the effect of this limitation by motivating more hospitals to conduct similar researches to create multicenter larger sample study in the future.

## Conclusion

We demonstrated disparities in baseline demographics, clinical characteristics, and hospital outcomes between AMI pilgrims and non-pilgrim patients. Although AMI pilgrims had less cardiovascular risk factors and were early revascularized, they showed higher rates of post myocardial infarction complication and poor hospital outcomes. The policy makers should take comprehensive, proper health coverage including implementation of pre-hajj screening and education programs, primary and secondary preventive measures, and proper awareness identifying those disparities into consideration.

## Data Availability

The data that support the findings of this study are available on reasonable request from corresponding author but not publicly available due to privacy.

## Abbreviations

AMI: Acute myocardial Infarction
BMI: Body mass index
CABG: Coronary artery bypass grafting
CAD: Coronary artery disease
CVA: Cerebrovascular accidents
CVD: Cardiovascular Disease
DBT: Door to balloon Time
DLP: Dyslipidemia
DM: Diabetes mellitus
HB: Hemoglobin
HBA1C: Glycosylated hemoglobin
HTN: Hypertension
IHD: Ischemic heart disease
KAMC: King Abdullah Medical City
LM: Left main
LOS: Length of stay
LVD: Left ventricular dysfunction
LVEF: Left ventricular ejection fraction
LVT: Left ventricular thrombus
MV: Mechanical ventilation
MVD: Multi vessel disease
NSTEMI: Non ST-elevation myocardial infarction
PCI: Percutaneous coronary intervention
PPCI: Primary percutaneous coronary intervention
STEMI: ST-elevation myocardial infarction
3VD: Three vessel disease
